# Rainfall variation and child health: effect of rainfall on diarrhea among under 5 children in Rwanda, 2010

**DOI:** 10.1186/s12889-016-3435-9

**Published:** 2016-08-05

**Authors:** Assumpta Mukabutera, Dana Thomson, Megan Murray, Paulin Basinga, Laetitia Nyirazinyoye, Sidney Atwood, Kevin P. Savage, Aimable Ngirimana, Bethany L. Hedt-Gauthier

**Affiliations:** 1 University of Rwanda School of Public Health, Kigali, Rwanda; 2 Department of Global Health and Social Medicine, Harvard Medical School, Boston, USA; 3 Epidemiology Departments, Harvard School of Public Health, Boston, USA; 4 Rwanda Biomedical Center (RBC), Kigali, Rwanda; 5 Division of Global Health Equity, Brigham and Women’s Hospital, Boston, USA; 6 Rwanda Meteorological Agency, Kigali, Rwanda; 7 Partners In Health, Kigali, Rwanda

**Keywords:** Diarrhea, Rainfall, Runoff, Climate change, Rwanda, DHS

## Abstract

**Background:**

Diarrhea among children under 5 years of age has long been a major public health concern. Previous studies have suggested an association between rainfall and diarrhea. Here, we examined the association between Rwandan rainfall patterns and childhood diarrhea and the impact of household sanitation variables on this relationship.

**Methods:**

We derived a series of rain-related variables in Rwanda based on daily rainfall measurements and hydrological models built from daily precipitation measurements collected between 2009 and 2011. Using these data and the 2010 Rwanda Demographic and Health Survey database, we measured the association between total monthly rainfall, monthly rainfall intensity, runoff water and anomalous rainfall and the occurrence of diarrhea in children under 5 years of age.

**Results:**

Among the 8601 children under 5 years of age included in the survey, 13.2 % reported having diarrhea within the 2 weeks prior to the survey. We found that higher levels of runoff were protective against diarrhea compared to low levels among children who lived in households with unimproved toilet facilities (OR = 0.54, 95 % CI: [0.34, 0.87] for moderate runoff and OR = 0.50, 95 % CI: [0.29, 0.86] for high runoff) but had no impact among children in household with improved toilets.

**Conclusion:**

Our finding that children in households with unimproved toilets were less likely to report diarrhea during periods of high runoff highlights the vulnerabilities of those living without adequate sanitation to the negative health impacts of environmental events.

## Background

Diarrhea is one of the top three causes of childhood mortality globally and remains a significant global health concern, with an estimated 2.5 billion cases occurring per year among children under five, more than half of which occur in Africa and South Asia [1]. Previous literature has suggested associations between rainfall patterns and diarrhea [2–6]. For example, a study in 14 sub-Saharan African countries including Rwanda reported an association between low rainfall and an increased risk of diarrhea [2]. Similar results were reported from Northern India where a study showed that reduced precipitation led to a 2–8 % increase in the incidence of diarrhea [3].

However little is known about other variables related to rainfall and their individual or collective effect on diarrhea incidence. In this study, we selected four rainfall variables that had been linked to the incidence of diarrhea in previous studies. First, we considered quantity of rain noting that this may be associated with diarrhea directly, as risk might be higher among individuals who have less water available [2, 4, 7, 8] and indirectly, as hygiene might be impaired if the quantity of rain is abnormally high or low [9]. We also considered runoff, a function of rainfall intensity and soil infiltration, which could contribute to environmental cleaning thereby reducing waterborne diseases [10, 11]. Finally, we considered unusually high or low rainfall compared to long-term (30 year) norms as this could affect diarrhea by forcing community members to alter their behavior [2, 12].

The impact of rainfall patterns on diarrhea is likely to be most extreme when sanitation is compromised [13]. In 2000, almost 1.73 million deaths due to diarrheal diseases were attributable to unsafe water, unimproved sanitation and poor hygiene; 68 % of these were in children and more than 99 % occurred in developing countries [9]. One study used 172 DHS datasets from 70 countries to show that improved water and sanitation infrastructure lowered the odds of children suffering from diarrhea by 7–17% [14]. Other studies from Guatemala [9], Benin [10] and Ecuador [11] demonstrate that household hygiene, access to clean water and sanitation all had a substantial impact on the risk of diarrhea in children. These studies suggest that the effect of rainfall variables on the occurrence of diarrhea may differ depending on household sanitation and water access.

Very few studies have explored the effect of rainfall and the potential modification of that effect by sanitation on the incidence of diarrhea in sub-Saharan Africa and to our knowledge, this has never been studied in Rwanda. In this analysis, we tested whether rainfall patterns affected the incidence of diarrhea in Rwanda among children under 5 years of age and whether this relationship varied with sanitation practices. A better understanding of the impact that rainfall has on diarrhea may help us to find ways to mitigate the risk of child illness and death [15].

## Methods

### Study design and population

We used individual-level data collected in the 2010 Rwanda Demographic and Health Survey (RDHS) and rainfall data from the Rwanda Meteorological Agency in the Rwanda Ministry of Infrastructure, the Famine Early Warning Systems (FEWS) Network and the US National Oceanic and Atmospheric Administration (NOAA) [16–19].

The RDHS is a cross-sectional survey conducted every 5 years by the Rwandan government with support from Independent Consulting Firm (ICF) International. In the 2010 RDHS, women aged 15 to 49 years answered detailed questions about themselves, their households and all of their children. Of the 13,790 women eligible, 13,671 (99.1 %) responded. The most common reasons for non-response were the woman not being home at the time of interview, the woman being incapacitated or her refusal to participate in the study. The 2010 RDHS used a two-stage sampling process. In the first stage, 492 villages were randomly selected with probability proportional to village size, stratified by district. In the second stage, 26 households from each village were randomly selected. A geographic (GPS) coordinate was collected in each village and randomly geo-displaced by up to 2 km for urban neighborhoods and 5 km for rural villages with one in every 100 rural coordinates displaced up to 10 km.

We obtained daily rainfall data collected at 14 stations across Rwanda between January 2009 and December 2011 from the Rwanda Meteorological Agency; 10-day estimates of the 30-year (1971–2000) long-term average rainfall for grid cells of approximately 30 km^2^ from the FEWS Network and monthly runoff estimates from NOAA which uses a global hydrological model for 30 km^2^ grid cells (described in [16]). We used the GPS data collected in the RDHS to determine the weather station and the 30 km^2^ grid closest in location and date to each household and assigned each child these rain-associated variables. Because household respondents in the same village were often interviewed over a period ranging from 2 days to 1 week, rainfall variables for children within a village did not vary.

#### Outcome assessment

In the 2010 RDHS, mothers were asked for each child under age 5 whether that child experienced one or more episodes of diarrhea during the 2 weeks preceding the survey.

#### Primary predictors: rainfall variables

We considered four aspects of rainfall that could impact the occurrence of diarrhea: total monthly rainfall, monthly rainfall intensity, runoff water and anomalous rainfall (Table 1). The first two variables, calculated from Rwanda Meteorological Agency data, were the *total monthly rainfall*, defined as the sum of the daily rainfall in last 30 days prior to the survey and *monthly rainfall intensity*, defined as the average daily rain for the month compared to the average daily rain for the year. While these two variables are correlated, the first variable is linked to the hypothesis that having more rain matters whereas the second is linked to the hypothesis that having more (or less) rain than usual matters. The third variable, *runoff water*, was the sum of the runoff from the NOAA database in the month prior to the date of data collection and finally, *anomalous rainfall* was defined as the rain for last 6 months compared to the long-term rainfall for the same 6 month window for the previous 30 years and calculated using FEWS data.

**Table 1:** Rainfall variable calculations and sources

Variable	Calculation	Data source	Possible interactions
Total monthly rain	Total rainfall in the 30 days prior to the survey: Sum of daily rainfall in last 30 days prior to the survey	RMI-MD	*Access to water*: *High rainfall (continuous pattern):* Individuals using surface or pump water may switch to rainfall water during periods of heavy rainfall especially if water is inaccessible. *High rainfall (downpour pattern)*: People using tap water may experience interruptions in water supply during heavy rains because silt in the water can clog filters at treatment plants.*Low rainfall:* Households that rely on surface water or tap water (treated surface water) sometimes experience water shortages during periods of no rain, especially during periods in which rain is expected.
			*Quality of drinking water*: *High rainfall (continuous or downpour pattern)*: Heavy rainfall may contaminate surface water by carrying waste and sediment into water sources used for drinking.*Low rainfall*: Low rainfall may force people to use contaminated water sources that they would not normally access.
			*Quality of toilet*: *High rainfall (continuous or downpour pattern):* With adequate water people may engage in more vigorous cleaning. *Low rainfall*: Very low rainfall (drought) may compromise cleaning and sanitation. *Quality of stool disposal*: *High rainfall (downpour pattern):* If stool is not disposed of adequately, heavy rain might wash away contaminated material away from the vicinity of the household. *High rainfall (continuous* *pattern):* If rainfall prevents normal mobility, stool may be disposed of nearer the household.
			*Shared toilet*: *High rainfall* (continuous or downpour pattern): Cleaning may be facilitated by the availability of water. *Low rainfall*: conversely, lack of water may prevent adequate sanitation.
Runoff water	Total run off in mm/month the previous month: The sum of the runoff in the month prior to the date of data collection.	NOAA	*Quality of drinking water*: Runoff water and flooding can lead to the contamination of surface water. Sediment in surface water can clog filters in water treatment plants and cause short-term interruptions in city water supply.
			*Quality of toilet*: Runoff water cleanse the environment of contaminating feces. However, low run off may create stagnant pools that foster the growth of harmful micro-organisms.
Monthly rainfall intensity	Rain for the month compared to the rain for the year: average daily rainfall for 30 days prior to the survey minus average daily rainfall for 365 days before the survey. A measure >0 means the month was wetter than the annual average.	*RMNR-MA*	*Access to water*: *Rainy season*: Individuals who use surface or pump water may use water from rainfall during wet months. Piped water may be interrupted during the rainy season because increased silt in the water can clog filters at the treatment plant. *Dry season*: Households that rely on surface water or tap water (treated surface water) sometimes experience water shortages during the dry season.
			*Quality of drinking water*: *Rainy season*: During the rainy season runoff and agitation by rain can decrease water quality by introducing silt and waste into surface water. Households that switch to rainwater during the rainy season, however, generally do not experience a decrease in water quality. *Dry season*: During the dry season, people who rely on surface water may experience a decrease in water quality.
			*Quality of toilet*: Good toilets are only effective if they are cleaned. *Dry season:* If the household is short of water, toilets may be less well cleaned.
			*Quality of stool disposal*: *Rainy season, drier annual average*: If it is a rainy time of year, some parents may not dispose of their children’s stools properly in latrines and rather dispose of stools directly into the environment close to the house.
Anomalous rainfall	The rain for last 6 months compared to the long-term rainfall in the corresponding 6 months for the previous 30 years: Sum of the daily rainfall for last 180 days minus sum of rainfall for full year from decadal estimates divided by two. A measure less than 0 mean that there was less rainfall for the 180 days than expected based on the long-term rainfall.	*RMNR-MA*, FEWS	*Access to water*: When water is available people may use it without looking for water from an improved source. Households plan their water storage and usage based on past experience of weather patterns, for example storing large quantities of water for the dry season and rationing it until the rains are expected again. If a rainy season does not produce as much water as expected, or if a dry season lasts longer than expected, water shortages may occur and result in poor hygiene, and under consumption of water.
			*Quality of toilet*: Families try to store enough water to get through expected dry seasons, though if the dry season is longer than expected or comes earlier than expected, families may face water shortages and reduce good hygiene practices, such as cleaning the toilet, because water is being used sparingly for other purposes.
			*Shared toilet*: If the rainfall is less or more than expected, families may face issues regarding their behaviors like hygiene practices, such as cleaning the shared toilet, because water is being used for other purposes or because the shared toilet is dirty due to mud from outside. Unexpected rain may influence increased need for water consumption or careless hygiene that may influence diarrhea transmission.

Key: *RMNR-MA* Rwanda Ministry of nature resources Meteorological Agency, *NOAA* [US] National Oceanic and Atmospheric Administration, *FEWS* Famine Early Warning System

To facilitate the interpretation of the results by decision and policy makers, rainfall variables were categorized into three levels. For the total monthly rainfall variable, we calculated a mean and standard deviation for the entire country. We classified total monthly rainfall as normal if it fell within one standard deviation of the mean, low if it was less than the mean by more than one standard deviation, and high if it was greater than the mean by more than one standard deviation. We used an identical process for categorizing the three remaining rainfall-related variables.

#### Potential confounders and effect modifiers: sanitation variables

We considered the following household demographic factors ascertained in the DHS as potential confounders of the rainfall-diarrhea relationship: child’s age, sex, use deworming medication in the last 6 months; mother’s age, employment status, and education level; and household’s urban/rural location, number of under-five children and wealth quintile. We also considered the following sanitation factors, as confounders or effect modifiers: easy access to water, defined as having water on the premises or obtainable within 30 min; quality of drinking water, considered “improved” if water was drawn from a protected spring, public tap, or standpipe and “unimproved” otherwise; quality of toilet, considered “improved” if toilets are pit latrines with a slab or flush toilets connected to a piped sewer system or septic tank and “unimproved” if they are pit latrines without a slab or open pits; stool disposal, considered to be “adequate” if the child used a toilet or latrine or if the fecal matter was placed/rinsed/poured into a toilet or latrine and inadequate otherwise; and shared toilet, considered shared if used by more than just the members of the household.

### Data analysis

We assessed the association between socio-demographic variables and diarrhea using Chi-squared tests. We used univariate logistic regression analysis to assess the association between household sanitation and rainfall variables and the outcome, diarrhea. All socio-demographic variables that were significant at the α = 0.1 level were included in the final multivariable logistic regression model. We constructed a multivariable logistic regression model to assess the adjusted impact of the four rainfall variables and diarrhea controlling for variables identified in the previous step. We selected the final model using manual backward stepwise regression. We first considered the rainfall-sanitation interaction terms and then the rainfall variables, removing variables one at a time until all remaining rainfall variables and interaction terms were significant at the α = 0.05 significance level. The analysis was completed in Stata v12 (StataCorpLP; 4905 Lakeway Drive, College Station, TX, USA), using survey commands to account for clustering of data, stratification and unequal probability of sampling.

## Results

Of 8601 children under age five included in the survey, 1132 (13.2 %) reported diarrhea in the 2 weeks prior to the survey. The mean age of children included in the survey was 31 months (SD = 17 months). Boys (51 %) and girls (49 %) were almost equally represented in the study; 79.3 % received treatment for intestinal parasites in the 6 months prior to the survey. The mothers’ mean age was 31 years (SD = 6.6 years). The majority (91 %) of mothers did not have a secondary education and most of them (79.6 %) worked outside the home. Most surveyed households (88 %) were located in rural areas and 61 % had at least one child younger than 5 years. Nearly half of the households (44.7 %) were in the lowest two wealth quintiles.

In the univariate analysis, children were more likely to report diarrhea within the previous 2 weeks if they were aged 12–23 months, were boys, had not received treatment for intestinal parasites or lived in wealthier households (Table 2). They were also more likely to report diarrhea if their mothers were under 18 years of age, did not work outside the home and had fewer than 4 children.

**Table 2:** Prevalence of diarrhea among children under 5 years of age from the 2010 DHS

Variable	Sample description			Reported diarrhea in previous 2 weeks		
	*N* (weighted)†	%	*n* (weighted)†	%	95 % CI	Chi-square *p*-value
Diarrhea	–		1132	13.2	[12.3,14.1]	N/A
Age in months (mean = 31, SD = 17)						<0.001
< 6	732	8.5	48	6.6	[5.0,8.6]	
6–11	841	9.8	184	21.8	[19.1,24.9]	
12–23	1616	18.8	404	25.0	[22.8,27.4]	
24–35	1824	21.2	242	13.3	[11.6,15.1]	
36–47	1739	20.2	152	8.7	[7.4,10.3]	
48–59	1849	21.5	103	5.6	[4.5,6.8]	
Gender						0.023
Boys	4361	50.7	610	14.0	[12.9,15.2]	
Girls	4240	49.3	522	12.3	[11.2,13.5]	
Treated for intestinal parasites within past 6 months						0.001
No	1783	20.7	275	15.4	[13.8,17.2]	
Yes	6813	79.3	856	12.6	[11.7,13.5]	
Mother’s age in years (mean = 31, SD = 6.6)						<0.001
15–24	1541	17.9	285	18.5	[16.3,20.9]	
25–34	4744	55.2	600	12.7	[11.6,13.7]	
35–49	2316	26.9	247	10.7	[9.3,12.2]	
Mother employed outside the home						0.022
No	1752	91.1	265	15.1	[13.1,17.4]	
Yes	6841	8.9	867	12.7	[11.8,13.6]	
Residence						0.746
Urban	1031	20.4	140	13.6	[11.0,16.6]	
Rural	7570	79.6	992	13.1	[12.2,14.1]	
Mother’s highest educational level						0.243
No education and primary	7840	12	1044	13.3	[12.4,14.3]	
Secondary and High	762	88	88	11.6	[9.2,14.5]	
Number of mother’s living children						<0.001
1–3 children	5212	60.6	751	14.4	[13.3,15.6]	
4+ children	3389	39.4	381	11.2	[10.2,12.4]	
Household in lowest 2 wealth quintiles						<0.001
No	4759	55.3	561	11.8	[10.7,12.9]	
Yes	3842	44.7	571	14.9	[13.6,16.2]	
Total	8601	100	1132	13.2	[12.3,14.1]	

†*N*(weighted) is the reported *N* accounting for the inverse probability sample weight

Children from households with easy access to water (less than 30 min) were less likely to report diarrhea than those further than 30 min from a water source as were children from households with inadequate stool disposal and those with shared toilets (Table 3). Children assessed during periods of high runoff were less likely to have reported diarrhea in the previous 2 weeks than those assessed during the period with low runoff water.

**Table 3:** Distribution of diarrhea by rainfall and household sanitation characteristics

Rainfall variables	*N* (weighted)†	% with diarrhea within subgroup	OR	95 % CI
Rainfall variables				
Total monthly rain				
Low	1769	13.5	1.00	
Average	5946	13.1	0.96	[0.79,1.17]
High	885	13.2	0.97	[0.96,12.5]
Runoff water				
Low runoff	1570	15.6	1.00	
Moderate runoff	5084	12.7	0.79	[0.62,1.00]
High runoff	1947	12.3	0.76	[0.59,0.98]
Monthly rainfall intensity				
Dry	1657	13.6	1.00	
Normal	5237	13.5	0.99	[0.81,1.20]
Wet	1706	11.7	0.84	[0.66,1.06]
Anomalous rainfall				
Below normal	1752	13.3	1.00	
Normal	5050	13.6	1.03	[0.83,1.26]
Above normal	1799	12.0	0.89	[0.70,1.12]
Household sanitation variables				
Time to get water				
≥ 30 min	4467	14.0	1.00	
< 30 min	4042	11.9	0.83	[0.73,0.96]
Source of drinking water				
Unimproved	2.385	14.3	1.00	
Improved	6128	12.5	0.86	[0.73,1.01]
Toilet quality				
Unimproved	2309	14.3	1.00	
Improved	6200	12.6	0.86	[0.72,1.03]
Adequate Stool disposal				
Yes	7092	12.5	1.00	
No	1405	16.9	1.43	[1.21,1.67]
Shared toilet				
No	6743	12.0	1.00	
Yes	1661	16.6	1.46	[1.25,1.60]

†*N*(weighted) is the reported *N* accounting for the inverse probability sample weight

In the multivariable analysis, we found that increased runoff was protective against diarrhea compared to low runoff (Table 4). None of the remaining rainfall factors were associated with diarrhea.

**Table 4:** Multivariable model results of the association between rainfall, household sanitation factors and child diarrhea prevalence

Predictors	Full model			Adjusted reduced model		
	Coefficient	95 % Confidence interval		Coefficient	95 % Confidence interval	
Rainfall variables						
Total monthly rain						
Low	Ref	–	–	–	–	–
Average	0.06	−0.18	0.31	–	–	–
High	0.10	−0.26	−0.47	–	–	–
Runoff water						
Low runoff	Ref	–	–	Ref	–	–
Moderate runoff	−0.61	−1.09	−0.14	−0.61	−1.07	−0.14
High runoff	−0.73	−1.29	−0.16	−0.70	−1.24	−0.15
Monthly rain intensity						
Dry	Ref	–	–	–	–	–
Normal	−0.04	−0.29	0.21	–	–	–
Wet	−0.10	−0.47	0.27	–	–	–
Anomalous rainfall						
Below normal	Ref	–	–	–	–	–
Normal	0.26	−0.12	0.64	–	–	–
Above normal	0.50	−0.06	1.06	–	–	–
Sanitation variables						
Time to get water						
≥ 30 min	Ref	–	–	–	–	–
< 30 min	−0.18	−0.33	−0.03	−0.18	−0.33	0.04
Household source of drinking water						
Unimproved source	Ref	–	–	–	–	–
Improved source	0.17	−0.20	0.55	−0.05	−0.22	1.13
Toilet quality						
Unimproved	Ref	–	–	–	–	–
Improved	−0.40	−0.83	0.03	−0.41	−0.85	0.02
Stool disposal						
Good disposal	Ref	–	–	–	–	–
Bad disposal	0.44	−0.26	0.62	0.44	0.26	0.62
Shared toilet						
No	Ref	–	–	–	–	–
Yes	0.34	0.17	0.50	0.34	0.17	0.50
Interaction term						
Runoff*toilet quality						
Moderate runoff*improved toilet	0.53	−0.05	1.01	0.55	0.07	1.03
High*Improved toilet	0.62	−0.05	1.19	0.61	0.04	1.18
Anomalous rainfall* toilet quality						
Normal rain*improved source of water	−0.20	−0.64	0.23	–	–	–
Above normal * improved source of water	−0.51	−1.07	0.04	–	–	–

Adjusted model: controlled for child’s age, sex, deworming, mother’s age, mother’s working status, number of under five children and wealth index

Further, when we examined the effect of the interaction terms, we found that the association between runoff and diarrhea was modified by toilet quality; more runoff was a protective factor only for children in households with unimproved toilet facilities (OR = 0.54, *p* = 0.010 for moderate runoff and OR = 0.50, *p* = 0.012 for high runoff) (Table 5); but had no impact among children in households with improved toilets.

**Table 5:** Effect of runoff water given the quality of toilet

Quality of toilet	Runoff water	OR	95 % CI
Unimproved toilet	Low runoff	Ref	
	Moderate runoff	0.54	[0.34, 0.87]
	High runoff	0.50	[0.29, 0.86]
Improved toilet	Low runoff	Ref	
	Moderate runoff	0.94	[0.74, 1.20]
	High runoff	0.92	[0.70, 1.21]

Adjusted model: controlled for child’s age, sex, deworming, mother’s age, mother’s working status, number of under five children, wealth index and sanitation factors (easy access to water, source of drinking water, quality of toilet, stool disposal and toilet sharing)

## Discussion

In this study of the association between rainfall variation and diarrhea among children under 5 years of age in Rwanda, we found that high runoff protected against diarrhea in children who used unimproved toilets but not in those whose homes had better sanitation. No other rainfall-related variables that we explored had a significant impact on diarrhea. Although the risk of diarrhea was affected by socio-demographic and sanitation determinants such as age, sex, mother’s age, occupation, wealth index, access to water, toilet sharing and stool disposal, adjustment for these factors did not alter the impact of runoff.

Previous studies on the association between rainfall and diarrhea demonstrate conflicting results. Some studies find that high runoff following rainfall increases the risk of diarrhea [4, 5, 15] while others show that runoff water protects against diarrhea [6, 13]. Our findings support this latter result, at least in Rwanda where much of the country is hilly and building codes prevent houses from being built in floodplains [20]. Following heavy rainfall, runoff water pools in valleys, usually fairly distant from homes. Indeed, the Rwanda DHS data show that only 10 % of Rwandans use surface water for drinking while most use water from rainfall, thus avoiding the pooled runoff [21]. Since most of the unimproved toilets in Rwanda are pit latrines located outdoors often on hillsides, it is possible that the runoff facilitates cleansing of the household environment. This interpretation is consistent with our finding that runoff water only reduced diarrhea risk in people with unimproved toilets. Knowing that one quarter of Rwandan households have unimproved toilet facilities and are at an increased risk of diarrhea under certain environmental conditions should contribute to the mounting evidence of the critical importance of improving access to quality toilet facilities in this setting.

Our study has several important limitations. Its cross-sectional nature, wherein data was collected at only one time point, limited our ability to address potential temporal confounders. While data were collected during the same time period within regions, data collection occurred at different time points across regions. Thus, the prevalence of diarrhea during the interview periods may have differed across regions due to variability in exposures to risk factors other than rainfall. Another limitation is the coarse granularity of the rainfall data. The long term rainfall measurements used to calculate the anomalous rainfall variable and the runoff variable was based on 30-by-30 km grid cell, therefore rainfall measurements assigned to specific households may be different from the actual values at the households. Furthermore, daily rainfall measures were from the closest weather station and thus may be different from the actual rainfall at households due to variability in local rainfall.

## Conclusion

While previous research has shown that there is an association between rainfall and diarrhea, this is the first research in Rwanda to explore the relationship between the households’ quantifiable rainfall variables on diarrhea prevalence among children under 5 years and to assess whether these effects vary by levels of sanitation. We found that increased runoff was associated with a decrease in childhood diarrhea. High runoff was a protective factor only for children in household with unimproved toilet facilities but had no impact among children in households with improved toilets. During periods of high runoff water, children in households with unimproved toilets were less likely to report diarrhea. These results emphasize the need for interventions aimed at improving household sanitation including construction of improved toilets.

## Abbreviations

CI, confidence interval; FEWS, the Famine Early Warning Systems; GPS, Global Positioning System; ICF, Independent Consulting Firm; NOAA, Network and the US National Oceanic and Atmospheric Administration; OR, odds ratio; RBC, Rwanda Biomedical Center; RDHS, Rwanda Demographic and Health Survey; SD, standard deviation; USA, United State of America
